# A global assessment of freshwater mollusk shell oxygen isotope signatures and their relation to precipitation and stream water

**DOI:** 10.1038/s41598-019-40369-0

**Published:** 2019-03-13

**Authors:** L. Pfister, C. Grave, J.-N. Beisel, J. J. McDonnell

**Affiliations:** 1 Luxembourg Institute of Science and Technology, Environmental Research and Innovation, Catchment and eco-hydrology research group, Esch-sur-Alzette, Luxembourg; 2 University of Luxembourg, Faculty of Science, Technology and Communication, Esch-sur-Alzette, Luxembourg; 3 Ecole Nationale du Génie de l’Eau et de l’Environnement de Strasbourg, Strasbourg, France; 4 Université de Strasbourg, CNRS, LIVE UMR 7362 Strasbourg, France; 5 Global Institute for Water Security, University of Saskatchewan, Saskatoon, SK Canada; 6 School of Resources and Environmental Engineering, Ludong University, Yantai, China; 7 School of Geography, Earth & Environmental Sciences, University of Birmingham, Birmingham, UK

## Abstract

Records of δ^18^O in stream flow are critical for understanding and modeling hydrological, ecological, biogeochemical and atmospheric processes. However, the number of such records are extremely limited globally and the length of such time series are usually less than a decade. This situation severely handicaps their use in model testing and evaluation. Here we present a global assessment of freshwater mollusk (bivalves & gastropods) isotope data from 25 river basins that have stream water isotope values, water temperature data and shell material isotope signatures. Our data span a latitude range of 37.50°S to 52.06°N. We show that δ^18^O signatures in freshwater mollusks are able to explain 95% of the variance of stream water δ^18^O. We use shell δ^18^O values and water temperature data to reconstruct stream water δ^18^O signatures. With freshwater mussel life expectancy ranging from a few years up to 200 years, this translation of mollusk metabolic properties into long term stream water isotope records is a promising approach for substantially extending global stream water isotope records in time and space.

## Introduction

Stream water stable isotopes of oxygen and hydrogen have been used for decades in hydrological process studies on water source and age^1,2^. However, the vast majority of these studies have been restricted to relatively short time periods (covering a few years at best) in small experimental catchments. Long stream water isotope records, while of great value for enhancing our understanding of the water cycle in river basins, or assessing environmental and climatic changes on the continental water cycle, remain restricted to only a few large river basins as linked to the Global Network of Isotopes in Rivers (GNIR) of the International Atomic Energy Agency^3–5^.

Recent work has suggested that freshwater mollusks have the potential to complement the scarce stream water isotope records as living archives of in-stream environmental conditions^6–10^. Freshwater mollusks live in a wide variety of aquatic habitats and potentially hold information in their successive growth bands on interannual fluctuations in stream water isotope signatures over multiple decades and even centuries. For rivers in India, δ^18^O signatures in freshwater mollusks have been shown to follow stream water δ^18^O across different topographic settings^10^. To date, no global assessment has been undertaken to see if mollusk isotope records match streamflow isotope records across different continents, elevations, climates and hydrological regimes (e.g. from rain to snow fed rivers).

We hypothesize that shell-related δ^18^O signatures are a strong proxy of δ^18^O in stream water across a wide range of climates and hydrological regimes – offering the potential for freshwater mollusks to reconstruct historical streamwater isotope signals in global rivers. We leverage the fact that oxygen isotopes in mollusk shell material precipitates in equilibrium with water and past studies that have shown it can serve as a robust proxy for water temperature reconstruction^11^.

Here we present a global assessment of mollusk shell δ^18^O isotope signatures and their corresponding precipitation and stream water δ^18^O isotope data. We base this on published oxygen isotope signatures obtained from growth bands of freshwater mollusk shells (bivalves & gastropods) collected in 25 river basins (33 sampling sites; ~100 analysed aragonitic mollusk shells) with contrasting elevations and climates.

## Results

From an initial list of 170 individual studies with published isotope signatures in freshwater mollusks, only 15 studies on 25 rivers and 33 sampling sites had both stream water isotope values and shell material isotope values. We relied on these 15 studies for conducting our comparative analysis (Table S1). For 22 rivers water temperature data was also available for a subsequent shell-based reconstruction of stream water δ^18^O signals. The mollusk sampling sites spanned a latitudinal range of 37.50°S to 52.06°N and an elevational range of 2 to 3250 m.a.s.l. Maximum elevations for the river basins ranged from 2 to 7620 m.a.s.l. and catchment areas ranged from ~75 km^2^ (Fleming Creek, South Carolina) to 400.000 km^2^ (Niger River in Africa).

**Figure 1:**
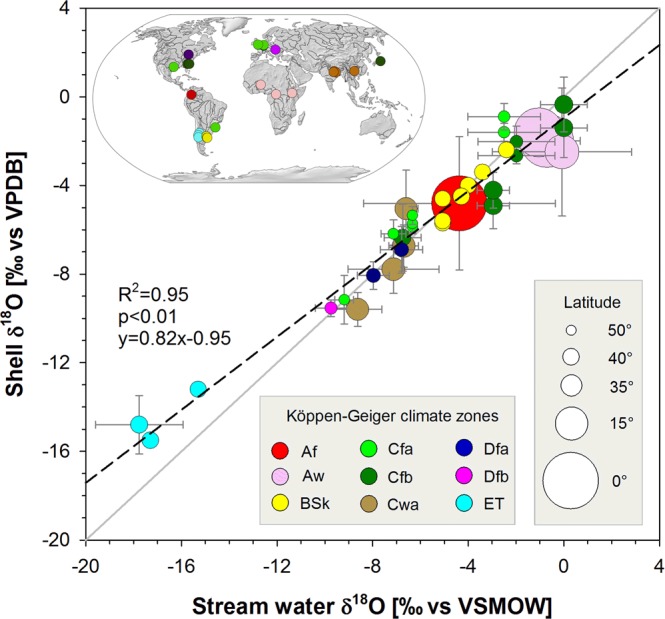
δ^18^O signals in stream water vs. freshwater mollusk shells [mean δ^18^O values with standard deviations] for 33 sampling sites across 25 river basins [map] and 9 Köppen-Geiger^12^ climate zones. Shell δ^18^O = oxygen isotopic composition of the carbonate, expressed as a deviation in ‰ from a standard carbonate, the VPDB (Vienna Pee Dee Belemnite). Water δ^18^O = oxygen isotopic composition of the water, expressed as a deviation in ‰ from the Vienna Standard Mean Ocean Water (VSMOW). Grey line: 1:1 line. Black dashed line: regression line (slope = 0.82). Colored dots: Köppen-Geiger^12^ climate zones in mollusk sampling locations ([Main climate–Precipitation–Temperature]; Af: Equatorial–fully humid; Aw: Equatorial–winter dry; BSk: Arid–summer dry–cold arid; Cfa: Temperate–fully humid–hot summer; Cfb: Temperate–fully humid–warm summer; Cwa: Temperate–winter dry–hot summer; Dfa: Cold–fully humid–hot summer; Dfb: Cold–fully humid–warm summer; ET: polar tundra. Dot size is proportional to the latitude of the shell and water sampling site. The map was created in ArcGIS version 10.5 (http://desktop.arcgis.com/) using free vector and raster map data [ne_50m_rivers_lake_centerlines; ne_110m_coastline; ne_110m_ocean; MSR_50M] made available by Natural Earth (http://naturalearthdata.com). All maps are in the public domain (http://www.naturalearthdata.com/about/terms-of-use/).

In most sites, the δ^18^O signatures (without correction for temperature effects) in mollusks mirrored the δ^18^O values found in stream water, with mean values plotting close to the 1:1 line (slope of regression line = 0.82) and standard deviations below 1.5‰ (Fig. 1; Table S2). Overall, shell δ^18^O explained 95% of the variance of stream water δ^18^O. As a corollary, the δ^18^O signatures in mollusks largely reflected δ^18^O values and latitudinal gradients in precipitation – here represented by δ^18^O ranges in precipitation across Köppen-Geiger^12^ climate zones proper to each river basin (Figs S2b, S2e; Table S2). Notwithstanding regional topographic effects, δ^18^O signatures in freshwater mollusks appear largely controlled by the climate conditions (as defined in the Köppen-Geiger^12^ classification) prevailing along a latitudinal gradient in the 25 river basins – spanning from equatorial to polar climates (Fig. 1). While such relations have been suggested at specific field sites^10^, our study is the first to show such a relationship across catchment sizes, latitudes, elevations and ecoregions.

Outliers to the general pattern included sites located at higher elevations [e.g. ET Köppen climate zone], where stream water would be slightly more depleted than shell material (Fig. 1). Alternatively, sites located at lower latitudes and elevations [e.g. Aw Köppen climate zone] had stream water that tended to be slightly more enriched in comparison to shell material. In some sites, reported δ^18^O values in streamflow and mollusks showed large variability (as expressed by their respective standard deviations in Table S2). The Amazon River for instance, showed a very strong altitudinal gradient between the basin’s headwaters located in the Andean mountains (5220 m.a.s.l.) and the mollusk sampling site (106 m.a.s.l.). This led to a pronounced seasonality in isotopic signatures, as well as a strong altitudinal control on the depletion effect in precipitation. We hypothesize that this in turn, may lead to a strong seasonality in both stream water δ^18^O values (snow melt) and mollusk shell δ^18^O signatures – as expressed in high standard deviations of both precipitation and stream water isotope signatures (3 and 4‰, respectively; Fig. 1 & Table S2). For the Niger River, mean δ^18^O values in stream water and mollusk shells differed by nearly 2.5‰ and standard deviations were close to 3‰ – reflecting contrasting within-watershed climate zones, ranging from gaining rivers (defined as flow increasing with basin area) in the humid rain fed headwaters, to losing sections (defined as flow losses with increasing basin area) near the Sahara^13^, finally leading into more humid regions and gaining flow further downstream.

Depending on the species and the prevailing climate (temperature) and environmental (e.g. salinity) conditions, bivalves may reduce or even cease their growth during more or less prolonged periods of time^14^. This may eventually lead to an increasing discrepancy between shell and stream water δ^18^O, with shell δ^18^O signals exhibiting truncated patterns across potentially reduced growth periods. As a corollary this may lead to larger differences between stream water and shell δ^18^O signals, as noticed for both low and high latitudes in our set of 25 river basins (Fig. 1). At low latitudes, amplitudes of seasonal stream water temperature are rather small, whilst mean annual water temperatures are high (Table S2; Fig. S2a). As an example, the Oubangui River (4.21°N lat.) has a mean annual water temperature of 28.6 °C and a seasonal amplitude of stream water temperature of 6.4 °C. The rather small variability in stream water temperature (standard deviation σ = 1.2 °C for the Oubangui River) could favor the overall representativeness of δ^18^O values measured in shells, even in case of intermittent growth periods (caused by very high stream water temperatures, or severe low flow conditions). Towards higher latitudes, amplitudes of stream water temperature tend to increase, whilst mean annual water temperatures decrease (Fig. S2a). The Huron River (42.33°N) has a mean annual water temperature of 14.2 °C and a seasonal amplitude of stream water temperature of 27 °C. The large variability in stream water temperature (σ = 9.2 °C for the Huron River) increases the probability of a reduced representativeness of δ^18^O values measured from shell growth bands excluding periods with water temperatures below a species-specific threshold^10,14^.

**Figure 2:**
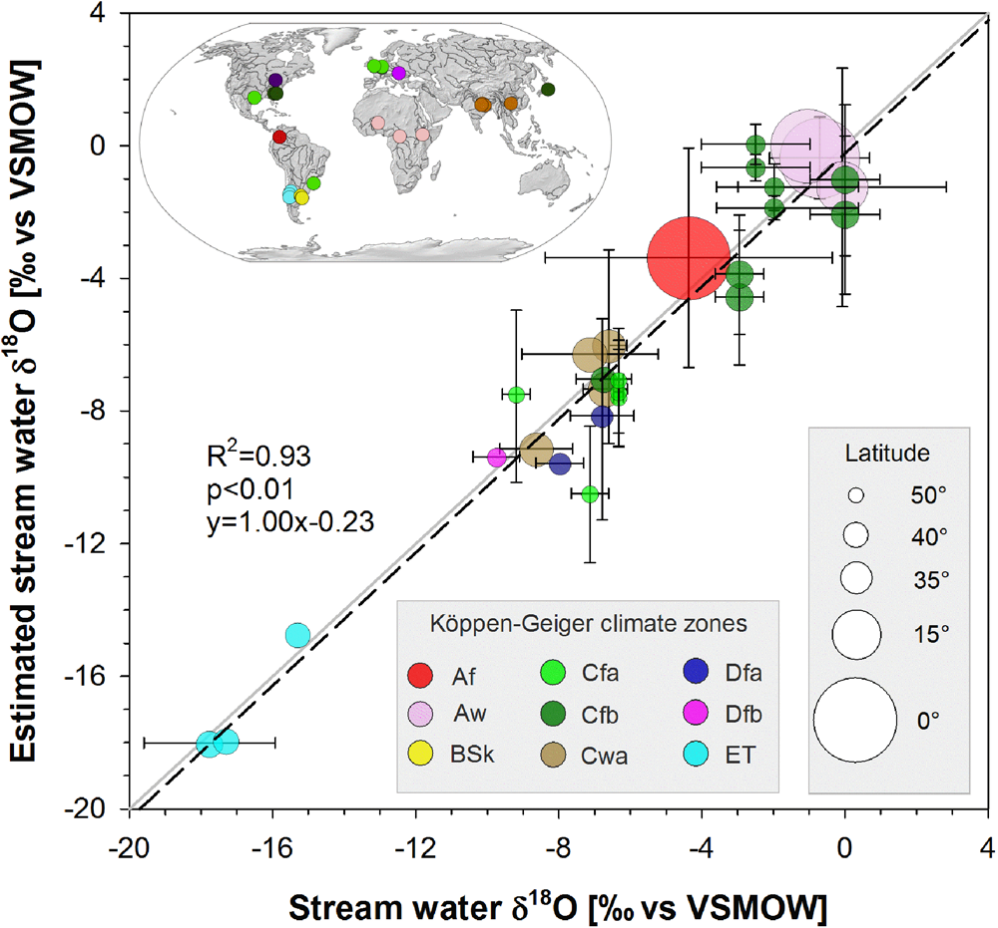
δ^18^O signals in stream water [mean δ^18^O values with standard deviations] and δ^18^O signals estimated (accounting for temperature effects) from shell δ^18^O values [mean δ^18^O values with standard deviations] for 22 river basins [map] and 9 Köppen-Geiger^12^ climate zones. Stream water δ^18^O = oxygen isotopic composition of the water, expressed as a deviation in ‰ from the Vienna Standard Mean Ocean Water (VSMOW). Grey line: 1:1 line. Black dashed line: regression line (slope = 1.00). Colored dots: Köppen-Geiger^12^ climate zones in mollusk sampling locations ([Main climate–Precipitation–Temperature]; Af: Equatorial–fully humid; Aw: Equatorial–winter dry; BSk: Arid–summer dry–cold arid; Cfa: Temperate–fully humid–hot summer; Cfb: Temperate–fully humid–warm summer; Cwa: Temperate–winter dry–hot summer; Dfa: Cold–fully humid–hot summer; Dfb: Cold–fully humid–warm summer; ET: polar tundra. Dot size is proportional to the latitude of the shell and water sampling site. The map was created in ArcGIS version 10.5 (http://desktop.arcgis.com/) using free vector and raster map data [ne_50m_rivers_lake_centerlines; ne_110m_coastline; ne_110m_ocean; MSR_50M] made available by Natural Earth (http://naturalearthdata.com). All maps are in the public domain (http://www.naturalearthdata.com/about/terms-of-use/).

Given the strong control of stream water temperature on mollusk shell growth, stream water δ^18^O and temperature are a common tool for reconstructing shell δ^18^O values^14,15^. We adapted this approach to reconstruct stream water δ^18^O for a subset of 22 rivers for which both shell mean δ^18^O and mean stream water temperature were available (Table S2). Our results show that estimated water δ^18^O closely mirrored δ^18^O values found in stream water (slope of the regression line = 1.00) – the reconstructed data explaining 93% of the variance of measured stream water δ^18^O (Fig. 2). These reconstructed stream water δ^18^O data reflected without any notable deviations, the difference in δ^18^O values of waters from different basins across a wide range of latitudes and climate settings (Table S2; Figs S2c & S2d). As expected, rivers at higher latitudes and/or elevations, with colder and wetter climates [e.g. ET Köppen climate zone], exhibited the most depleted reconstructed and measured isotope signatures; also as expected, stream water temperatures were lowest (mean annual ~5 °C) and δ^18^O values most depleted (~17‰ to ~15‰) in the glacier and snowmelt Andean rivers of the Mendoza province (Argentina; lat. 35–37°S). At lower latitudes the warm and drier climate conditions [e.g. Aw Köppen climate zone] signals were more enriched in reconstructed and measured isotope signatures in stream water (Figs 2; S2c,d). In African river basins, stream water temperatures were very high (e.g. Oubangui River at ~29 °C) and δ^18^O signatures were among the most enriched (e.g. ~0‰ in the Niger River) of our dataset (Table S2). When accounting for the variability in stream water temperature (as expressed through ± 1σ) and shell δ^18^O (±1σ) for calculating stream water δ^18^O (Fig. S2d), we found the smallest range of uncertainty for river basins located at low latitudes (e.g. ±1.2‰ for the Oubangui River). In river basins located at higher latitudes the range of uncertainty in δ^18^O was much larger (e.g. ±3‰ for the Huron River). A notable exception to this pattern is the Niger River, where a considerable uncertainty (±3.6‰) is triggered by the large variability in shell δ^18^O (σ = 2.89‰), despite a rather small seasonal stream water temperature amplitude (12.6 °C). The Amazon River equally exhibits a large uncertainty in estimated stream water δ^18^O (±3.3‰), with the effect of a small seasonal stream water amplitude (6.2 °C) outweighed by a large variability in shell δ^18^O (σ = 3.01‰).

## Discussion

Our global assessment of mollusk shell δ^18^O data across a latitudinal sequence of 33 sampling sites reveals the strong links between precipitation, stream water and shell signatures – suggesting the potential for freshwater mollusks to serve as archives of past isotopic signatures in rivers over a wide range of hydro-climatological settings. Long records of δ^18^O series are of great value for gaining a better understanding of long-term variability and/or non-stationary hydrological, ecological, biogeochemical and atmospheric responses to global change. However, the numbers of such records remain extremely limited globally and the length of such time series are usually less than a decade. An estimated 1000 freshwater bivalve species (order Unionoida) populate a large variety of river systems and lakes around the globe. Freshwater mussels are long-lived organisms, living an average of 10 years – with many species living 20 to 30 years and some up to two centuries^16^ (e.g. *Margaritifera margaritifera*)^17^. Such high longevity gives them the potential for recording across their successive growth bands long-term changes in environmental conditions. While past work has used mollusks for paleo-temperature reconstructions^18^, our analysis shows that mollusk shells formed in isotopic equilibrium with the surrounding water^11^ may serve as new tools for reconstructing stream water δ^18^O signatures over several decades from δ^18^O data series measured across mollusk shell increments.

In our global assessment of mollusk shell δ^18^O data, four studies relied on whole-shell analyses for determining time-averaged δ^18^O values. Another eleven studies had analyzed successive growth increments, delivering either seasonal or inter-annual variability in δ^18^O signals over multiple years. A limitation to the full reconstruction of stream water δ^18^O data is the sensitivity of freshwater mussels to stream water temperature or salinity that may induce growth periods of different lengths depending on latitude, elevation and prevailing climate conditions. Aestivation and growth interruption may eventually cause a limited representativeness of shell δ^18^O data for reconstruction of annual or inter-annual stream water δ^18^O signatures^19^. Various studies on freshwater bivalves have shown that shell growth ceases at temperatures below 12 °C^10,14,20^. At high latitudes/elevations, shell δ^18^O signals exhibit an increasingly truncated sinusoidal pattern with narrow peaks (essentially due to shell growth cessation in winter) and wide troughs^7^. At lower latitudes/elevations, elevated water temperatures (20 to 35 °C) may substantially modify metabolic rates in freshwater mollusks and compromise biological processes such as survival, growth and reproduction^21^. In tropical regions with typically small temperature variations the dominant control on shell δ^18^O variations are the changes in stream water δ^18^O or monsoonal influences^9,22^. High discharge or low water conditions may lead to more or less prolonged growth gaps, extending up to 150 days in the Oubangui and Niger river basins^9^.

An additional source of uncertainty relates to the water sampling protocols applied in the 33 mollusk collection sites used in our study. While water had been sampled at, or nearby shell collection sites, sampling frequency differed strongly – ranging from unique grab samples of water to fortnightly water sampling protocols extending over several years.

The highly variable temporal resolution in shell δ^18^O (time-averaged vs. successive growth increments) and river water δ^18^O (grab samples vs. continuous sampling) across our set of 33 sampling sites resulted in substantial differences between the related standard deviations of observed and reconstructed δ^18^O values. Ultimately, water and mollusk shell sampling frequency, as well as individual mollusk species’ physiology, ecology and life cycle are crucial for the interpretation of observed and reconstructed isotope signatures^23^.

When considering the effects of mollusk shell analysis for reconstructing historical data series, it is also important to consider that aragonite is thermodynamically unstable – with a gradual recrystallization to calcite leading to a ‘resetting’ of isotopic signatures^24^. A thorough mineralogical assessment of the degree of preservation of aragonitic mollusk shells is therefore strongly recommended (e.g. via X-ray diffraction)^25^. An important next step in this context is the implementation of calibration studies – either via controlled experiments or field surveys – contributing to a better understanding of the relationships between stable isotope signals and ecological and/or environmental variables. This will eventually lead to an improved interpretation of measured variability as ecological and/or environmental ‘signal’ or stochastic ‘noise’^26^.

Regardless of current limitations, δ^18^O records from mollusk shells have the potential to open up new research avenues for quantifying climate change impacts on the long-term isotope time series of precipitation and stream water^27^, leading to new mechanistic understanding of processes controlling water flow and quality^28^, or serving for the calibration and validation of flow and transport models^29,30^.

## Methods

We used the search terms SHELL, ISOTOP*, FRESHWATER and OXYGEN in Web of Science and found 170 individual studies on isotope signatures in mollusks. This list was reduced to 15 studies carried out on 25 rivers (lakes were ruled out), for which stable isotope data was available for both stream water and shell material (from 28 different freshwater mollusk species; Fig. S1; Table S1). Stream water temperature data was extracted from the retained list of studies, except for the regions of Northwestern Mendoza, Central-eastern Mendoza and Southern Mendoza, for which water temperature data was taken from Scheibler *et al*.^31^.

The protocols used for mollusk shell preparation, shell material sampling and isotope analyses slightly differed among the 15 studies (Table S3). Four out of the 15 studies relied on analytical protocols that consisted in crushing one or several mollusk shells and subsequent δ^18^O analysis of the collected shell material via a mass spectrometer (Table S3). In eleven studies shell annual growth bands were sampled either with micromilling devices, dental drills, or scalpel blades. The collected shell material was then analyzed with a mass spectrometer. Secondary Ion Mass Spectrometry has been recently used for obtaining high-resolution records of isotope signatures in mollusk shells^32–34^. Prior to isotope analysis, growth bands are identified through dyeing with Mutvei’s solution.

For the 33 mollusk sampling sites of our global assessment, δ^18^O values were either integrated (i.e. obtained via whole-shell analyses) or spanning over successive growth increments (i.e. years of growth bands). For an individual site, mean values either correspond to an average of δ^18^O values from several shells or to an average of δ^18^O values sampled across shell growth increments.

For converting δ^18^O in shell material to δ^18^O in stream water, the reported studies relied on equations relating water temperature and δ^18^O in stream water (e.g. Dettman *et al*.^14^, Gonfiantini *et al*.^15^, Friedman and O’Neil^35^), such as: 

1 $$1000\,{\rm{ln}}\alpha =2.559({10}^{6}{{\rm{T}}}^{-2})-0.715$$

where T = stream water temp. (in °K) and α = fractionation between water and aragonite

2 $${{\rm{\alpha }}}_{{\rm{water}}}^{{\rm{aragonite}}}=\frac{[1000+{{\rm{\delta }}}^{18}{{\rm{O}}}_{{\rm{ar}}}({\rm{VSMOW}})]}{[1000+{{\rm{\delta }}}^{18}{{\rm{O}}}_{{\rm{w}}}({\rm{VSMOW}})]}\,,\,{\rm{where}}\,ar\,{\rm{is}}\,{\rm{shell}}\,{\rm{aragonite}}\,{\rm{and}}\,w\,{\rm{is}}\,{\rm{water}}$$

Since δ^18^O_ar_ values are first made relative to the Vienna Pee Dee Belemnite (VPDB) reference, they are commonly converted to the Vienna Standard Mean Ocean Water (VSMOW) as per Gonfiantini *et al*.^15^:

3 $${{\rm{\delta }}}^{18}{{\rm{O}}}_{{\rm{ar}}}({\rm{VSMOW}})={{\rm{\alpha }}}_{{\rm{water}}}^{{\rm{aragonite}}}(1000+{{\rm{\delta }}}^{18}{{\rm{O}}}_{{\rm{ar}}}({\rm{VPDB}}))\mbox{--}1000$$

On the basis of equations (1) and (2) we were able to estimate stream water δ^18^O from shell δ^18^O and stream water temperature:

4 $${{\rm{\delta }}}^{18}{{\rm{O}}}_{{\rm{w}}}({\rm{VSMOW}})=\frac{[1000+{{\rm{\delta }}}^{18}{{\rm{O}}}_{{\rm{ar}}}({\rm{VSMOW}})]}{{{\rm{\alpha }}}_{{\rm{water}}}^{{\rm{aragonite}}}}-1000$$

We extracted the isotopic values in stream water and mollusk shells and stream water temperature data from the primary literature via the data extraction software PlotDigitizer, as well as from tables (Tables S1–S3).

For the 25 river basins, we relied on the Köppen-Geiger climate zones as per Rubel and Kottek^12^ to determine δ^18^O ranges in precipitation (Table S2 and Fig. S2).

## Supplementary information

Supplementary information

## Data Availability

The datasets are available from the individual studies retained for this work, as well as from the authors of this manuscript upon request.
