# Supplementary material for: A global assessment of freshwater mollusk shell oxygen isotope signatures and their relation to precipitation and stream water
Source: Sci Rep. 2019 Mar 13;9:4312. doi: 10.1038/s41598-019-40369-0 (PMC6416266; doi:10.1038/s41598-019-40369-0)
Supplement: Supplementary file 1 — Supplementary information [file 41598_2019_40369_MOESM1_ESM.pdf]

SUPPLEMENTAL MATERIAL

A global assessment of freshwater mollusk shell oxygen isotope signatures and their relation to precipitation and stream water

L. Pfister[1,2,*], C. Grave[3], J.-N. Beisel[3,4], J.J. McDonnell[5,6,7]

[1]Luxembourg Institute of Science and Technology, Environmental Research and Innovation, Catchment and eco-hydrology research group, Esch/Alzette, Luxembourg

[2]University of Luxembourg, Faculty of Science, Technology and Communication, Esch/Alzette, Luxembourg

[3]Ecole Nationale du Génie de l'Eau et de l'Environnement de Strasbourg, Strasbourg, France

[4]Université de Strasbourg, CNRS, LIVE UMR 7362, Strasbourg, France

[5]Global Institute for Water Security, University of Saskatchewan, Saskatoon SK Canada

[6]School of Resources and Environmental Engineering, Ludong University, Yantai, China

[7]School of Geography, Earth & Environmental Sciences, University of Birmingham, Birmingham, UK

[*] Corresponding author

Supplemental material

Biology of mollusk shells

Mollusks have been used extensively as environmental sentinel organisms and archives – extending paleotemperature records on the basis of shell growth rates and $\delta^{18}O$ signatures[11]. A limitation and source of uncertainty in the use of mollusk shell paleorecords of environmental signatures has been related to the irregular growth rates[16]. The shell material is produced by the mollusk's mantle as long as the environmental conditions remain favorable. In the case of excessively high or low water temperatures or water pollution, the mantle withdraws from the edges of the shell and the mollusk stops producing shell material.

As soon as shell growth resumes, a new growth band is formed and gradually covers the previous one. Darker and thinner bands are assumed to be produced during winter months[17]. Based on their growth bands, it is possible to estimate the age of a mollusk[17]. The average lifespan of freshwater mussels is largely species dependent – freshwater pearl mussels (*Margaritifera margaritifera*) living up to 200 years[16,17].

On $\delta^{18}O$ signatures in freshwater mollusks and their use in environmental research

While records of isotopic signatures in precipitation are available at global scale and over many decades, historical data on stream water isotope signatures is still patchy. Similar situations of limited data on past environmental conditions are typically overcome by using tree rings or sediment layers as natural archives – leading to the reconstruction of temperature chronicles or trends in soil and air chemical composition[36,37].

For overcoming the very limited availability of long stream water $\delta^{18}O$ series, we rely here on organisms living in freshwater bodies and potentially assimilating for decades the seasonal fluctuations in $\delta^{18}O$ of stream water. Mollusks precipitate their calcium carbonate shell in equilibrium with the surrounding water[14,18,38]. The oxygen isotopic composition of calcium carbonate in a mollusk's shell thus depends on water temperature (controlling the fractionation process) and water isotopic composition. As a consequence, mollusks record isotope signatures in stream water[39,40].

While the results from our global assessment are encouraging, more work needs to be done on the extraction of $\delta^{18}O$ values from mollusk shells and within-shell trends in isotopic amplitudes and averages and how this affects extracted isotope profiles from successive years[18]. Secondary Ion Mass Spectrometry (SIMS) may be a promising technique for future global testing of mollusks as a hydrological isotope recorder as shown by Linzmeier et al.[32] and Pfister et al.[34].

Sampling locations

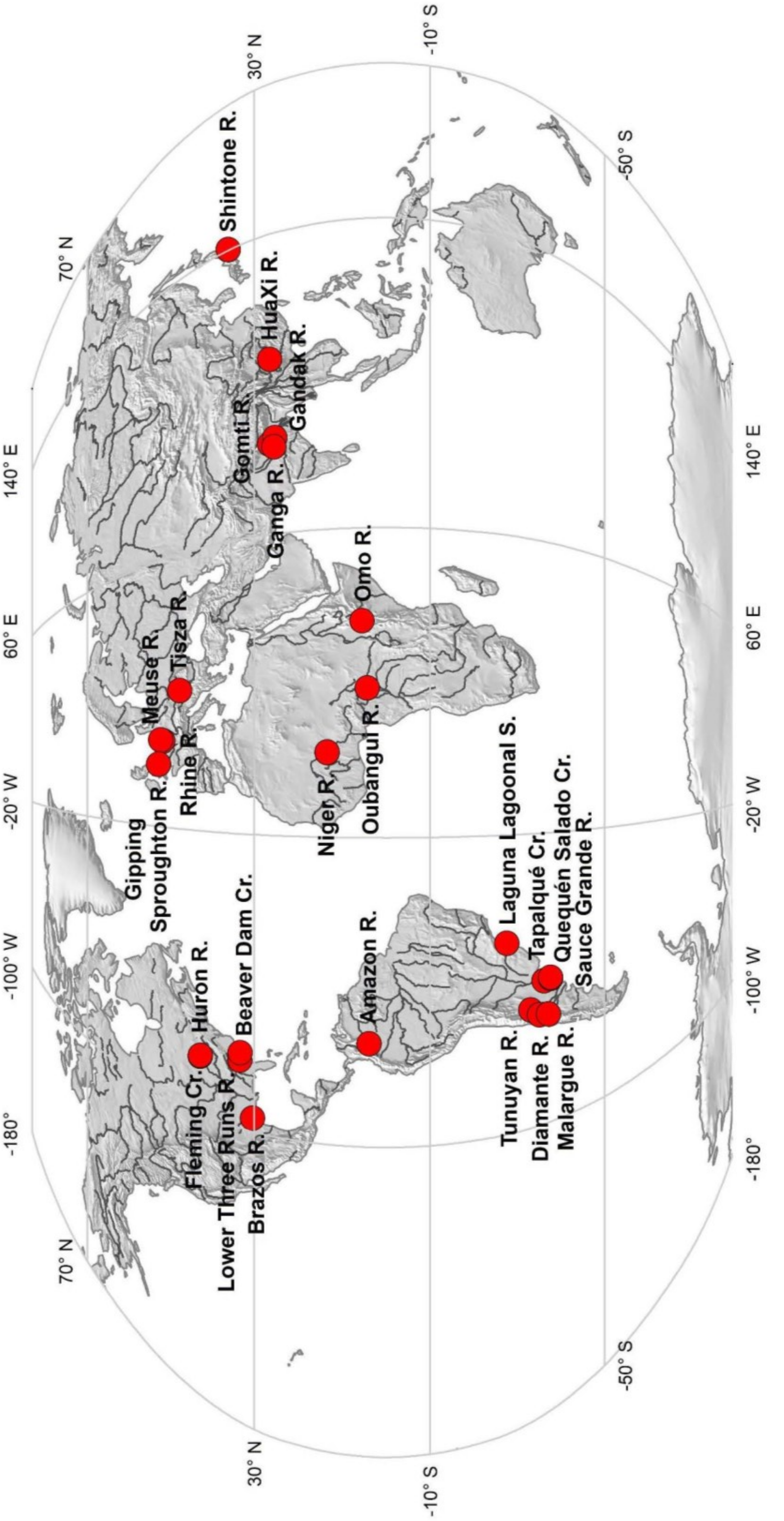

Figure S1. World map of studies on isotope signatures in freshwater mollusks. The map was created in ArcGIS version 10.5 (http://desktop.arcgis.com/) using free vector and raster map data [ne_50m_rivers_lake_centerlines; ne_110m_coastline; ne_110m_ocean; MSR_50M] made available by Natural Earth (http://naturalearthdata.com). All maps are in the public domain (http://www.naturalearthdata.com/about/terms-of-use/).

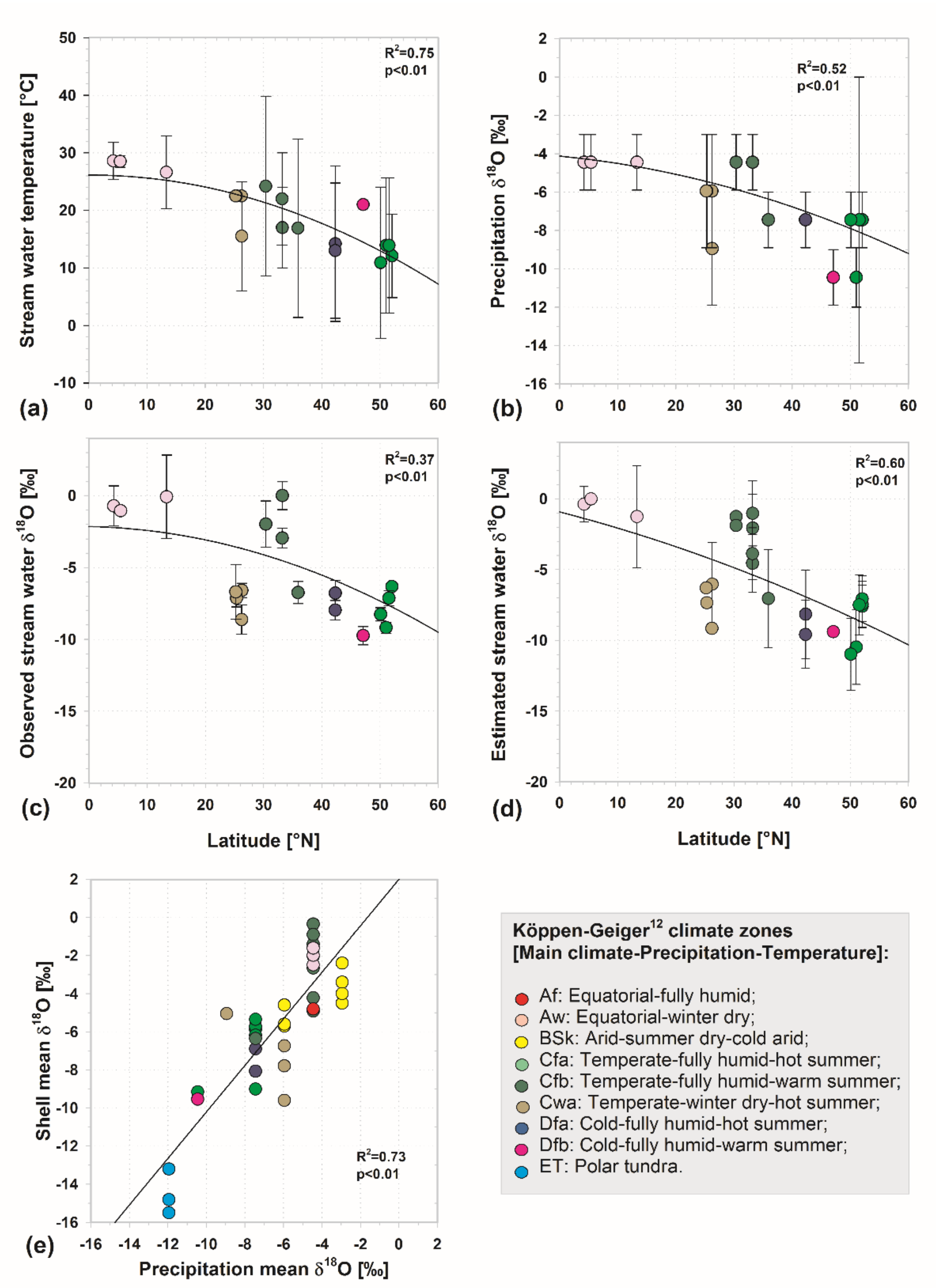

Figure S2. Latitudinal gradients in the Northern Hemisphere in (a) Stream water mean annual temperature [dots] and temperature amplitude [vertical bars], (b) mean annual $\delta^{18}O$ values in precipitation [dots] with minimum and maximum $\delta^{18}O$ values [vertical bars], (c) $\delta^{18}O$ values in stream water [mean $\delta^{18}O$ values with standard deviations], (d) mean estimated $\delta^{18}O$ values in stream water [mean $\delta^{18}O$ values with standard deviations]. (e) Mean annual $\delta^{18}O$ values in precipitation vs. mean $\delta^{18}O$ values in aragonitic bivalve shells. Shell $\delta^{18}O$ = oxygen isotopic composition of the carbonate, expressed as a deviation in ‰ from a standard carbonate, the VPDB (Vienna PeeDee Belemnite). Precipitation and stream water $\delta^{18}O$ = oxygen isotopic composition of the water, expressed as a deviation in ‰ from the Vienna Standard Mean Ocean Water (VSMOW).

Inventory of mollusk shell studies, sampling sites and species specifications

Authors	Site	Mollusk species	Class	Stream water	Shell isotope data	Collection date	Shell sampling site	
							Lat.	Elev.
Waghorne et al.[41]	Gipping Sproughton	*Bithynia tentaculata*	Gastropoda	2006-2007 (bi-monthly)	15 shells	2006-2007	52.06°N	~35 m
		Sphaerium corneum	Bivalvia		10 shells			
		Valvata piscinalis	Gastropoda		10 shells			
Versteegh et al.[7]	Rhine River (Netherlands)	*Unio tumidus*	Bivalvia	1997 – 2005 (at Lobith)	1 shell 1996-2003	2003 (Waal at Herwijnen)	51.55°N	~7 m
		Anodonta anatina	Bivalvia		1 shell 1996-2003			
	Meuse River (Netherlands)	*Unio pictorum*	Bivalvia	1997-2005 (at Eijden)	1 shell 1997-2005	2005 (near Kerkdriel)	51.05°N	~2 m
Demény et al.[42]	Tisza River (Hungary)	*Unio* sp.	Bivalvia	03/2001 - 12/2001 (every 1 to 2 weeks)	5 shells (3 to 8 samples from each shell)	collected 03/2001	47.1°N	91 m
Dettman et al.[14]	Huron River (Michigan, USA)	*Lampsilis ovata ventricosa*	Bivalvia	09/1990 - 11/1991 (twice per month)	1 shell 11/1990 - 05/1992 (49 samples)	collected 06/1992 (4 years old)	42.33°N	265 m
	Fleming Cr. (Michigan, USA)	*Alasmidonta viridis*	Bivalvia	03/1992 - 06/1992 (twice per month)	1 shell 11/1990 - 05/1992 (36 samples)	collected 10/1992 (6 years old)	42.30°N	256 m
Yoshimura et al.[43]	Shintone River, Honshu (Japan)	*Hyriopsis* sp.	Bivalvia	06/2005 - 12/2006 (monthly)	1 shell 2001 - 10/2007 (83 samples)	Collected 25/10/2007 (7 years old)	35.92°N	~2 m.
Caroll et al.[20]	Lower Three Runs, South Carolina (USA)	*Elliptio*	Bivalvia	1998-2002	2 shells 1998-2002 (50 samples per shell)	collected 06/2003 (5 & 10 years old)	33.22°N	63 m.
	Beaver Dam Creek, South Carolina (USA)	*Elliptio*	Bivalvia	1998-2002	2 shells 1998-2002 (50 samples per shell)	collected 06/2003 (12 & 6 years old)	33.19°N	200 m
Van Plantinga & Grossman[44]	Brazos River (Texas, USA)	*Amblema plicata*	Bivalvia	01/2012 – 01/2013 (weekly)	1 shell	09/08/2013	30.37°N	58 m
		Cyrtonaias tampicoensis	Bivalvia		1 shell			
Yan et al.[45]	HuaXi River	*Corbicula fluminea*	Bivalvia	01.2006 – 01.2007	2 shells	09.01.2007	26.26°N	1200 m
Gajurel et al.[10]	Gandak River (India)	*Bellamya bengalensis*	Gastropoda	2000-2001 (3 samples)	1 shell (28 samples)	2001	26.21°	67 m
	Gomti River (India)	*Bellamya bengalensis*	Gastropoda	2000-2001 (3 samples)	5 shells (lumped values per shell)	08/2001	25.34°N	74 m
	Ganga River (India)	*Bellamya bengalensis*	Gastropoda	2000-2004 (8 samples)	1 shell (31 samples)	2001	25.22°N	38 m
Kelemen et al.[9]	Niger River (Niger)	*Aspatharia dahomeyensis*	Bivalvia	03/2011 - 03/2013 (every 2 weeks)	1 shell 2011-2013 (64 samples)	collected 04/2013 (2 years old)	13.31°N	205 m
	Oubangui River (Central African Rep.)	*Chambardia wissmanni*	Bivalvia	08/2009-12/2013	several shells 01/2010 - 12/2012 (181 samples)	collected 03/2013	4.21°N	369 m

Table S1: Studies on isotope signatures in stream water and aragonitic shells of freshwater bivalves.

Authors	Site	Mollusk species	Class	Stream water	Shell isotope data	Collection date	Shell sampling site	
							Lat.	Elev.
Vonhof et al.[46]	Omo River (Kenya)	*Etheria elliptica*	Bivalvia	08/2004	1 shell (~29 samples)	2003 (2 years old)	5.41°N	381 m
Kaandorp et al.[22]	Amazon River (Peru)	*Anodontites trapesialis*	Bivalvia	09/1998-10/1999	09/1998 - 10/1999 (26 samples)	collected 21/10/1999	3.75°S	106 m
Colonese et al.[47]	Laguna Lagoonal System (Brazil)	*Anomalocardia flexuosa*	Bivalvia	08/2008-08/2009	2 shells (44 samples)	2007	27.5°S	~1 m
De Francesco and Hassan[48]	Northwestern Mendoza (Tunuyán River)	*Chilina mendozana*	Gastropoda	Panarello & Dapeña (1996)	17 shells	~2012	33-37°S	~900-1200m
		Heleobia hatcheri	Gastropoda					
	Central-eastern Mendoza (Diamante and Atuel Rivers)	*Heleobia kuesteri*	Gastropoda	Vogel et al. (1975)				~1200-3250m
		Heleobia parchappii	Gastropoda					
	Southern Mendoza (Atuel and Malargue Rivers)	*Lymnaea viator*	Gastropoda	Ostera & Dapeña (2003)				
		Pisidium chiquitanum	Bivalvia					
Bonadonna et al.[49]	Arroyo Tapalqué	*Chilina sp.*, *Littoridina sp.*	Gastropoda	17/10/93	3 shells	~1993	36°S-37.5°S	~40m
	Arroyo Quequén Salado			28/10/93 19/03/92	3 + 2 shells		37.5°S	~10m
	Sauce Grande			22/10/93	3 + 2 shells		37.5°S	~140m

Table S1: Studies on isotope signatures in stream water and aragonitic shells of freshwater bivalves (continued).

River	Köppen climate zones	Precipitation	Stream water							Shell			Reconstructed stream water	
			Temperature [°C]				δ^{18}O [‰]			δ^{18}O [‰]			δ^{18}O [‰]	
		Mean δ^{18}O [‰]	T°	n	σ	Min / Max	Mean	n	σ	Mean	n	σ	Mean	Upper/lower limit
Gipping R.	Cfb	-8.90 to -6.00	12.1	12	5.4	5 / 19.5	-6.33	6	0.14	-5.88	15	0.30	-7.59	-6.14 / -9.11
Gipping R.	Cfb	-8.90 to -6.00	12.1	12	5.4	5 / 19.5	-6.33	6	0.14	-5.72	10	0.41	-7.43	-5.87 / -9.06
Gipping R.	Cfb	-8.90 to -6.00	12.1	12	5.4	5 / 19.5	-6.33	6	0.14	-5.35	10	0.40	-7.06	-5.51 / -8.68
Meuse R.	Cfb	-8.90 to -6.00	13.9	71	6.7	2.5 / 26	-7.13	104	0.52	-6.18	42	0.62	-7.50	-5.49 / -9.61
Rhine R.	Dfc, Cfb	-14.9 to -6.00	13.9	49	7.0	2.5 / 26	-9.19	26	0.39	-9.16	80	1.09	-10.48	-7.94 / -13.12
Tisza R.	Dfb	-11.90 to -9.00	21.0*	1	-	-	-9.74	95	0.64	-9.54	5	0.38	-9.39	-
Huron R.	Dfa to Dfb	-8.90 to -6.00	14.2	34	9.2	2 / 29	-6.79	23	0.89	-6.90	41	1.06	-8.16	-5.22 / -11.28
Fleming Cr.	Dfa to Dfb	-8.90 to -6.00	13.0	32	7.9	2 / 25.5	-7.97	18	0.67	-8.07	13	0.63	-9.58	-7.31 / -11.99
Shintone R.	Cfa	-8.90 to -6.00	16.9	60	9.0	2 / 33	-6.74	19	0.77	-6.36	83	1.51	-7.05	-3.74 / -10.52
Lower Three Runs	Cfa	-5.90 to -3.00	17.0	43	5.0	9 / 23	~0	43	0.97	-1.4	53	1.34	-2.07	0.29 / -4.48
Lower Three Runs	Cfa	-5.90 to -3.00	17.0	43	5.0	9 / 23	~0	43	0.97	-0.35	43	1.23	-1.02	1.23 / -3.32
Beaver Dam Cr.	Cfa	-5.90 to -3.00	22.0	44	5.0	13 / 29	-2.95	44	0.68	-4.91	26	1.04	-4.56	-2.55 / -6.62
Beaver Dam Cr.	Cfa	-5.90 to -3.00	22.0	44	5.0	13 / 29	-2.95	44	0.68	-4.22	59	0.81	-3.87	-2.09 / -5.70
Brazos R.	Cfa	-5.90 to -3.00	24.2	20	7.6	6.7 / 37.8	-1.98	20	1.61	-2.03	14	0.70	-1.25	-0.55 / -1.95
Brazos R.	Cfa	-5.90 to -3.00	24.2	20	7.6	6.7 / 37.8	-1.98	20	1.61	-2.66	17	0.36	-1.88	-1.52 / -2.24
HuaXi R.	Cwa to Cwb	- 11.9 to -6.00	15.5	53	5.6	6 / 25	-6.60	51	0.50	-5.05	34	1.74	-6.03	-3.14 / -8.99
Gandak R.	Cwa to Cwb	-8.90 to -3.00	22.5	2	-	15 / 32	-8.63	3	1.02	-9.6	28	0.76	-9.15	-
Ganga R.	Cwa to Cwb	-8.90 to -3.00	22.5	2	-	15 / 32	-7.13	8	1.91	-7.79	31	1.09	-6.29	-
Gomti R.	Cwa to Cwb	-8.90 to -3.00	22.5	2	-	15 / 32	-6.7	3	0.61	-6.74	5	0.96	-7.34	-
Niger R.	Aw to Bsh	-5.90 to -3.00	26.6	50	3.7	18.5 / 31.1	-0.08	50	2.91	-2.49	64	2.89	-1.25	2.34 / -4.85
Oubangui R.	Aw	-5.90 to -3.00	28.6	91	1.2	25.1 / 31.5	-0.71	111	1.40	-1.99	181	1.01	-0.37	0.86 / -1.61
Omo R.	Aw to As	-5.90 to -3.00	28.5	2	-	27.5 / 29.5	-1.05	2	-	-1.61	29	0.78	-0.01	-
Amazon R.	Af	-5.90 to -3.00	27.6	23	1.6	25 / 31.2	-4.37	23	4.01	-4.81	57	3.01	-3.38	-0.07 / -6.70
Laguna Lagoonal System	Cfa	-5.90 to -3.00	21.0	25	3.9	14 / 30	-2.50	104	1.52	-1.6	-	0.4	-0.66	-0.26 / -1.06
Laguna Lagoonal System	Cfa	-5.90 to -3.00	21.0	25	3.9	14 / 30	-2.50	104	1.52	-0.9	44	0.6	0.04	0.64 / -0.56
North-Western Mendoza	ET	-14.90 to -9.00	5.4*	2	-	-	-17.77	3	1.83	-14.81	9	1.32	-18.01	-
Central-Eastern Mendoza	ET	-14.90 to -9.00	8.8*	6	-	-	-17.30	1	-	-15.50	1	-	-17.92	-
Southern Mendoza	ET	-14.90 to -9.00	12.9*	3	-	-	-15.40	1	-	-13.20	1	-	-14.73	-
Tapalqué Cr.	BSk	-5.90 to 0.00	-		-	-	-4.29	1	-	-4.50	1	-	-	-
Quequén Salado R.	BSk	-5.90 to 0.00	-		-	-	-4.41	2	-	-3.40	1	-	-	-
Quequén Salado R.	BSk	-5.90 to 0.00	-		-	-	-4.41	2	-	-4.00	1	-	-	-
Quequén Salado R.	BSk	-5.90 to 0.00	-		-	-	-4.41	2	-	-2.40	1	-	-	-
Sauce Grande R.	BSk	-8.90 to -3.00	-		-	-	-5.07	1	-	-4.60	1	-	-	-
Sauce Grande R.	BSk	-8.90 to -3.00	-		-	-	-5.07	1	-	-5.70	1	-	-	-
Sauce Grande R.	BSk	-8.90 to -3.00	-		-	-	-5.07	1	-	-5.60	1	-	-	-

Table S2: River basins, Köppen-Geiger climate zones (as per Rubel & Kottek[12]), Range in precipitation δ^{18}O (as per Terzer et al.[50]), Mean annual stream water temperature (except for values marked with an asterisk, taken from Scheibler et al.[31]), Mean values and standard deviations of δ^{18}O in stream water and aragonitic bivalve shells, Mean values with upper and lower bounds (for 1σ in water temperature and shell δ^{18}O) of reconstructed stream water δ^{18}O. n = number of observations.

Authors	Shell analyses	Isotope data source and extraction
Waghorne et al.[41]	Crushed shell, Mass spec. analyses	Average and standard deviation values directly taken from text (no raw data provided in article).
Versteegh et al.[7]	Shell growth bands, Micromill, Mass spec. analyses	Meuse River: Stream water (article Figure 4) and shell isotope data (article Figure 11-B) extracted via PlotDigitizer.
		Rhine River: Stream water (article Figure 4) and shell isotope data (article Figure 12-B) extracted via PlotDigitizer.
Demény et al.[42]	Shell growth bands, Dental drill, Mass spec. analyses	Stream water taken from Table 3 and shell isotope data (article Figure 1) extracted via PlotDigitizer.
Dettman et al.[14]	Shell growth bands, Micromill, Mass spec. analyses	Huron River: Stream water (article Figure 2) and shell isotope data (article Figure 5) extracted via PlotDigitizer.
		Fleming Creek: Stream water (article Figure 2) and shell isotope data (article Figure 6) extracted via PlotDigitizer.
Yoshimura et al.[43]	Shell growth bands, Dental drill, Mass spec. analyses	Stream water (article Figure 3) and shell isotope data (article Figure 5) extracted via PlotDigitizer.
Caroll et al.[20]	Shell growth bands, Micromill, Mass spec. analyses	Lower Three Runs: Stream water (article Figure 4) and shell isotope data (article appendix) extracted via PlotDigitizer.
		Beaver Dam Creek: Stream water (article Figure 4) and shell isotope data (article appendix) extracted via PlotDigitizer.
Van Plantinga & Grossman[44]	Shell growth bands, Micromill, Mass spec. analyses	Stream water isotope data and temperatures extracted via PlotDigitizer (article Figure 3A). Shell isotope data taken from Table 3.
Yan et al.[45]	Shell growth bands, Scalpel blade, Mass spec. analyses	HuaXi River: Stream water temperature and isotope data (article Figure 3) and shell isotope data (article Figure 4) extracted via PlotDigitizer.
Gajurel et al.[10]	Crushed shells & growth bands, Micromill, Mass spec. analyses	Gandak River: Stream water isotope data taken from article Table 2 and shell isotope data taken from article Table 4.
		Gomti River: Stream water isotope data taken from article article Table 2 and shell isotope data taken from Table 3.
		Ganga River: Stream water isotope data taken from article Table 2 and shell isotope data (article Figure 9) extracted via PlotDigitizer.
Kelemen et al.[9]	Shell growth bands, Micromill, Mass spec. analyses	Niger River: Stream water (article Figure 2) and shell isotope data (Appendix; Shell N37B) extracted via PlotDigitizer.
		Oubangui River: Stream water (article Figure 2) and shell isotope data (article Figure 6) extracted via PlotDigitizer.
Vonhof et al.[46]	Shell growth bands, Micromill, Mass spec. analyses	Omo River: Stream water taken from article Table 2 and shell isotope data (article Figure 4) extracted via PlotDigitizer.
Kaandorp et al.[22]	Shell growth bands, Micromill, Mass spec. analyses	Amazon River: Stream water taken from article Table 1 and shell isotope data taken from article Table 2.
Colonese et al.[47]	Shell growth bands, Micromill, Mass spec. analyses	Stream water taken from article discussion section and shell isotope data taken from article results section 3.4.
De Francesco & Hassan[48]	Crushed shells, Mass spec. analyses	North-western Mendoza: stream water isotope data taken from Panarello & Dapeña (1996) and shell isotope data taken from article Table 4.
		Central Eastern Mendoza: stream water and shell isotope data from article Table 4.
		Southern Mendoza: stream water and shell isotope data from article Table 4.
Bonadonna et al.[49]	Crushed shells, Mass spec. analyses	Stream water taken from Table 2 and shell isotope data taken from Table 1.

Table S3: Shell material analyses techniques and stream water and shell isotope data sources for the 15 selected studies.

References

[7]Versteegh, E., Troelstra, S., Vonhof, H. & Kroon, D. Oxygen isotope composition of bivalve seasonal growth increments and ambient water in the rivers Rhine and Meuse. PALAIOS **24**, 497-504 (2009).

[9]Kelemen Z. et al. Calibration of hydroclimate proxies in freshwater bivalve shells from Central and West Africa. *Geochimica et Cosmochimica Acta* **208**, 41-62 (2017).

[10]Gajurel, A.P., France-Lanord, C., Huyghe, P., Guilmette, C. & Gurung, D. C and O isotope compositions of modern fresh-water mollusc shells and river waters from the Himalaya and Ganga plain. *Chemical Geology* **233**, 156-183 (2006).

[11]Urey, H.C. The thermodynamic properties of isotopic substances. *Journal of the Chemical Society*, 562-581 (1947).

[12]Rubel, F. & Kottek, M. Observed and projected climate shifts 1901–2100 depicted by world maps of the Köppen-Geiger climate classification. *Meteorologische Zeitschrift* **19**, 135-141 (2010).

[14]Dettman, D.L., Reische, A.K. & Lohmann, K.C. Controls on the stable isotope composition of seasonal growth bands in aragonitic fresh-water bivalves (unionidae). *Geochimica et Cosmochimica Acta* **63**, 1049-1057 (1999).

[16]Anthony, J.L., Kesler, D.H., Downing, W.L. & Downing, J.A. Length-specific growth rates in freshwater mussels (Bivalvia: Unionidae): extreme longevity or generalized growth cessation? *Freshwater Biology* **46**, 1349-1359 (2001).

[17]Dunca, E., Söderberg, H. & Norrgrann, O. Shell growth and age determination in the freshwater pearl mussel *Margaritifera margaritifera* in Sweden: natural versus limed streams. *Ferrantia* **64**, 48-58 (2011).

[18]Goodwin, D.H., Schöne, B.R. & Dettman, D.L. Resolution and fidelity of oxygen isotopes as paleotemperature proxies in bivalve mollusk shells: models and observations. *PALAIOS* **18**, 110-125 (2003).

[20]Caroll, M., Romanek, C. & Paddock, L. The relationship between the hydrogen and oxygen isotopes of freshwater bivalve shells and their home streams. *Chemical Geology* **234**, 211-222 (2006).

[22]Kaandorp, R.J.G. Seasonal stable isotope variations of the modern Amazonian freshwater bivalve *Anodontites trapesialis*. *Palaeogeography, Palaeoclimatology, Palaeoecology* **194**, 339-354 (2003).

[31]Scheibler, E.E., Claps, M.C. & Roig-Juñent, S.A. Temporal and altitudinal variations in benthic macroinvertebrate assemblages in an Andean river basin of Argentina. *Journal of Limnology* **73**, 92-108 (2014).

[32]Linzmeier, B. J., Kozdon, R., Peters, S.E. & Valley, J.W. Oxygen isotope variability within Nautilus shell growth bands. *PLoS ONE* **11**, e0153890 (2016).

[34]Pfister, L. et al. Freshwater pearl mussels as a stream water stable isotope recorder. *Eco-hydrology*, e2007 (2018).

[36]Carrer, M. Individualistic and time-varying tree-ring growth to climate sensitivity. *PLoS ONE* **6**, e22813 (2011).

[37]Aymerich, I.F., Oliva, M., Giralt, S. & Martín-Herrero, J. Detection of tephra layers in Antarctic sediment cores with hyperspectral imaging. *PLoS ONE* **11**, e0146578 (2016).

[38]Schöne, B.R., Goodwin, D.H., Flessa, K.W., Dettman, D.L. & Roopnarine, P.D. Sclerochronology and growth of the bivalve mollusks *Chione (Chionista) fluctifraga* and *Chione (Chionista) cortezi* in the Gulf of California, Mexico. *Veliger* **45**, 45–54 (2002).

[39]Krantz, D.E., Williams, D.F. & Jones, D.S. Ecological and paleoenvironmental information using stable isotope profiles from living and fossil molluscs. *Palaeogeography, Palaeoclimatology, Palaeoecology* **58**, 249-266 (1987).

[40]Yan, H., Chen, J. & Xiao, J. A review on bivalve shell, a tool for reconstruction of paleo-climate and paleo-environment. *Chinese Journal of Geochemistry* **33**, 310-315 (2014).

[41]Waghorne, R., Hancock, J.D.R. & Candy, I. Environmental controls on the $\delta^{18}O$ composition of freshwater calcite and aragonite in a temperate, lowland river system: significance for palaeoclimatic studies. *Proceedings of the Geologists' Association* **123**, 576–583 (2012).

[42]Demény, A. et al. Stable isotope compositions and trace element concentrations in freshwater bivalve shells (*Unio* sp.) as indicators of environmental changes at Tiszapüspöki, eastern Hungary. *Central European Geology* **55**, 441–460 (2012).

[43]Yoshimura, T., Nakashima, R., Suzuki, A., Tomioka, N. & Kawahata, H. Oxygen and carbon isotope records of cultured freshwater pearl mussel *Hyriopsis* sp. shell from Lake Kasumigaura, Japan. *Journal of Paleolimnology* **43**, 437-448 (2015).

[44]Van Plantinga, A.A. & Grossman, E. Stable and clumped isotope sclerochronologies of mussels from the Brazos River, Texas (USA): Environmental and ecologic proxy. *Chemical Geology* **502**, 55-65 (2018).

[45]Yan, H., Lee, X., Zhou, H., Cheng, H., Peng, Y. & Zhou, Z. Stable isotope composition of the modern freshwater bivalve *Corbicula fluminea*. *Geochemical Journal* **43**, 379-387 (2009).

[46]Vonhof, H.B. et al. Environmental and climatic control on seasonal stable isotope variation of freshwater molluscan bivalves in the Turkana Basin (Kenya). *Palaeogeography, Palaeoclimatology, Palaeoecology* **383–384**, 16-26 (2013).

[47]Colonese, A.C. et al. Shell sclerochronology and stable isotopes of the bivalve *Anomalocardia flexuosa* (Linnaeus, 1767) from southern Brazil: Implications for environmental and archaeological studies. *Palaeogeography, Palaeoclimatology, Palaeoecology* **484**, 7-21 (2017).

[48]De Francesco, C.G. & Hassan, G.S. Stable isotope composition of freshwater mollusk shells from central-western Argentina. *Revista Brasileira de Paleontologia* **16**, 213-224 (2013).

[49]Bonadonna, F.P., Leone, G. & Zanchetta, G., 1999. Stable isotope analyses on the last 30 ka molluscan fauna from Pampa grassland, Bonaerense region, Argentina. *Palaeogeography, Palaeoclimatology, Palaeoecology* **153**, 289–308 (1999).

[50]Terzer, S., Wassenaar, L.I., Araguás-Araguás, L.J. & Aggarwal, P.K. Global isoscapes for $\delta^{18}O$ and $\delta^{2}H$ in precipitation: improved prediction using regionalized climatic regression models. *Hydrology and Earth System Sciences* **17**, 4713-4728 (2013).
